# Supplementary figures and images for: Impact of human mobility and networking on spread of COVID-19 at the time of the 1st and 2nd epidemic waves in Japan: An effective distance approach
Source: PLoS One. 2022 Aug 11;17(8):e0272996. doi: 10.1371/journal.pone.0272996 (PMC9371261; doi:10.1371/journal.pone.0272996)

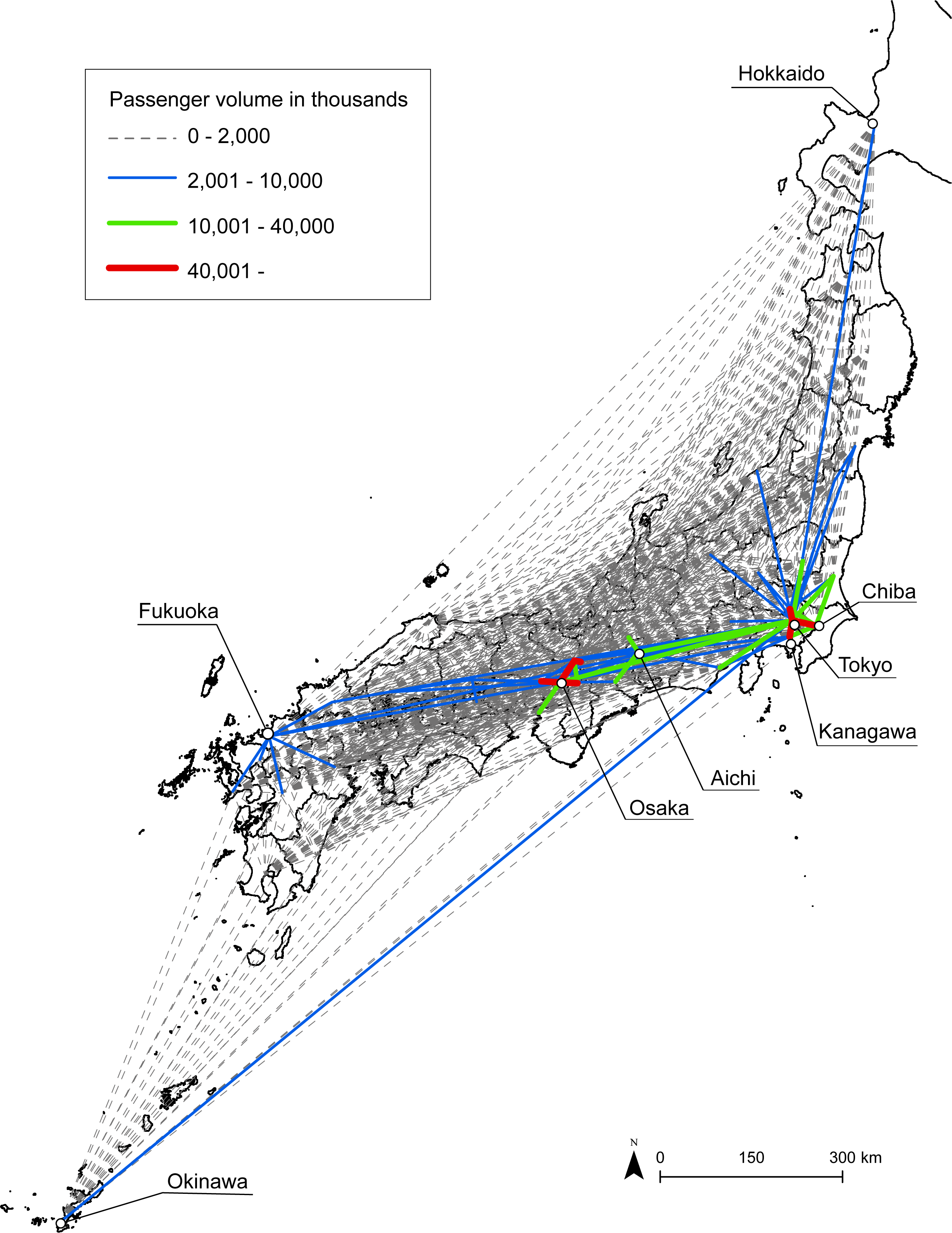

Supplement: S1 Fig — (TIF) [file pone.0272996.s001.tif]
