# Supplementary material for: Impact of human mobility and networking on spread of COVID-19 at the time of the 1st and 2nd epidemic waves in Japan: An effective distance approach
Source: PLoS One. 2022 Aug 11;17(8):e0272996. doi: 10.1371/journal.pone.0272996 (PMC9371261; doi:10.1371/journal.pone.0272996)
Supplement: S1 Table — (DOCX) [file pone.0272996.s002.docx]

| **Prefecture name** | **Total no. of cases at 1^st^ epidemic wave** | **Total no. of cases at 2^nd^ epidemic wave** |
| --- | --- | --- |
| **Hokkaido** | 1,054 | 719 |
| **Aomori** | 27 | 8 |
| **Iwate** | 0 | 19 |
| **Miyagi** | 88 | 116 |
| **Akita** | 16 | 33 |
| **Yamagata** | 69 | 9 |
| **Fukushima** | 81 | 77 |
| **Ibaraki** | 168 | 373 |
| **Tochigi** | 64 | 238 |
| **Gunma** | 149 | 288 |
| **Saitama** | 1,000 | 2,901 |
| **Chiba** | 797 | 1,772 |
| **Tokyo** | 5,197 | 15,572 |
| **Kanagawa** | 1,332 | 3,583 |
| **Niigata** | 82 | 60 |
| **Toyama** | 227 | 158 |
| **Ishikawa** | 295 | 329 |
| **Fukui** | 122 | 100 |
| **Yamanashi** | 60 | 113 |
| **Nagano** | 76 | 180 |
| **Gifu** | 150 | 405 |
| **Shizuoka** | 74 | 406 |
| **Aichi** | 510 | 3,993 |
| **Mie** | 45 | 323 |
| **Shiga** | 100 | 344 |
| **Kyoto** | 358 | 1,090 |
| **Osaka** | 1,781 | 6,710 |
| **Hyogo** | 699 | 1,571 |
| **Nara** | 91 | 424 |
| **Wakayama** | 63 | 167 |
| **Tottori** | 3 | 19 |
| **Shimane** | 24 | 113 |
| **Okayama** | 25 | 120 |
| **Hiroshima** | 166 | 291 |
| **Yamaguchi** | 37 | 130 |
| **Tokushima** | 5 | 125 |
| **Kagawa** | 28 | 50 |
| **Ehime** | 80 | 33 |
| **Kochi** | 74 | 51 |
| **Fukuoka** | 666 | 3,903 |
| **Saga** | 47 | 190 |
| **Nagasaki** | 17 | 214 |
| **Kumamoto** | 48 | 463 |
| **Oita** | 60 | 85 |
| **Miyazaki** | 17 | 341 |
| **Kagoshima** | 11 | 350 |
| **Okinawa** | 143 | 1,980 |
