# Supplementary material for: Impact of human mobility and networking on spread of COVID-19 at the time of the 1st and 2nd epidemic waves in Japan: An effective distance approach
Source: PLoS One. 2022 Aug 11;17(8):e0272996. doi: 10.1371/journal.pone.0272996 (PMC9371261; doi:10.1371/journal.pone.0272996)
Supplement: S2 Table — (DOCX) [file pone.0272996.s003.docx]

| **Prefecture name** | **Time of arrival [day] at 1^st^ epidemic wave** | **Time of arrival [day] at 2^nd^ epidemic wave** |
| --- | --- | --- |
| **Hokkaido** | 13 | 0 |
| **Aomori** | 68 | NA |
| **Iwate** | NA | NA |
| **Miyagi** | 45 | 47 |
| **Akita** | 51 | 79 |
| **Yamagata** | 76 | NA |
| **Fukushima** | 52 | 84 |
| **Ibaraki** | 62 | 48 |
| **Tochigi** | 38 | 33 |
| **Gunma** | 52 | 70 |
| **Saitama** | 17 | 23 |
| **Chiba** | 15 | 28 |
| **Tokyo** | 9 | 0 |
| **Kanagawa** | 0 | 0 |
| **Niigata** | 45 | 64 |
| **Toyama** | 75 | 66 |
| **Ishikawa** | 37 | 73 |
| **Fukui** | 63 | 87 |
| **Yamanashi** | 51 | 66 |
| **Nagano** | 41 | 60 |
| **Gifu** | 42 | 50 |
| **Shizuoka** | 44 | 50 |
| **Aichi** | 11 | 49 |
| **Mie** | 15 | 58 |
| **Shiga** | 50 | 57 |
| **Kyoto** | 15 | 31 |
| **Osaka** | 14 | 30 |
| **Hyogo** | 46 | 40 |
| **Nara** | 13 | 40 |
| **Wakayama** | 29 | 56 |
| **Tottori** | 86 | NA |
| **Shimane** | 85 | NA |
| **Okayama** | 67 | 50 |
| **Hiroshima** | 52 | 46 |
| **Yamaguchi** | 48 | 70 |
| **Tokushima** | 41 | 60 |
| **Kagawa** | 62 | NA |
| **Ehime** | 47 | NA |
| **Kochi** | 45 | NA |
| **Fukuoka** | 36 | 35 |
| **Saga** | 58 | 56 |
| **Nagasaki** | 59 | 46 |
| **Kumamoto** | 38 | 56 |
| **Oita** | 48 | 70 |
| **Miyazaki** | 49 | 58 |
| **Kagoshima** | 71 | 37 |
| **Okinawa** | 30 | 55 |
